# Should ecologists prefer model‐ over distance‐based multivariate methods?

**DOI:** 10.1002/ece3.6059

**Published:** 2020-02-14

**Authors:** Jonathan F. Jupke, Ralf B. Schäfer

**Affiliations:** ^1^ iES Landau Institute for Environmental Sciences University Koblenz‐Landau Landau Germany

**Keywords:** multivariate analysis, numerical simulations, ordination, statistical models, variable selection

## Abstract

Ecological data sets often record the abundance of species, together with a set of explanatory variables. Multivariate statistical methods are optimal to analyze such data and are thus frequently used in ecology for exploration, visualization, and inference. Most approaches are based on pairwise distance matrices instead of the sites‐by‐species matrix, which stands in stark contrast to univariate statistics, where data models, assuming specific distributions, are the norm. However, through advances in statistical theory and computational power, models for multivariate data have gained traction. Systematic simulation‐based performance evaluations of these methods are important as guides for practitioners but still lacking. Here, we compare two model‐based methods, multivariate generalized linear models (MvGLMs) and constrained quadratic ordination (CQO), with two distance‐based methods, distance‐based redundancy analysis (dbRDA) and canonical correspondence analysis (CCA). We studied the performance of the methods to discriminate between causal variables and noise variables for 190 simulated data sets covering different sample sizes and data distributions. MvGLM and dbRDA differentiated accurately between causal and noise variables. The former had the lowest false‐positive rate (0.008), while the latter had the lowest false‐negative rate (0.027). CQO and CCA had the highest false‐negative rate (0.291) and false‐positive rate (0.256), respectively, where these error rates were typically high for data sets with linear responses. Our study shows that both model‐ and distance‐based methods have their place in the ecologist's statistical toolbox. MvGLM and dbRDA are reliable for analyzing species–environment relations, whereas both CQO and CCA exhibited considerable flaws, especially with linear environmental gradients.

## Graphical abstract

Despite their great potential and flexibility, model‐based multivariate statistical methods are rarely used in ecological studies. We compared the performances of two algorithm‐based and two model‐based multivariate methods in discerning between true explanatory variables and noise variables in simulated community abundance data sets. Our results show pronounced performance differences among and between model‐ and algorithm‐based methods and highlight multivariate generalized linear models and distance‐based redundancy analysis as valuable methods.

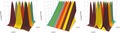

## 1 INTRODUCTION

Which environmental gradients determine species abundances and community composition is one of the most essential questions in ecology (Clements, 1907) and the current alteration of ecosystems at an unprecedented rate endows it with a new urgency (Pacifici et al., 2015). Given the complexity of simulating ecological systems under artificial conditions (e.g., in microcosms), monitoring the abundance or occurrence of taxa across sites with variable environmental conditions has been one approach to tackle this question. Related studies deliver a sites‐by‐species matrix **Y** containing multivariate species abundances, which is then statistically related to a sites‐by‐predictors matrix **X**, containing information on the environmental predictors. From a statistical perspective, **Y** has many undesirable properties such as intercorrelations between variables, for example, through biotic interactions (Morales‐Castilla, Matias, Gravel, & Araújo, 2015), probability distributions other than the normal, more species than sites (high dimensionality, especially in DNA barcoding studies, Cristescu, 2014), and many zeros, because most species are commonly absent from most sites (sparsity, McGill et al., 2007).

While univariate data (i.e., one response but possibly multiple explanatory variables) are routinely analyzed by model‐based methods such as ANOVA, generalized linear models, and linear mixed models, multivariate data are most often analyzed with distance‐based methods. The latter analyze a pairwise matrix of distances or dissimilarities instead of the sites‐by‐species matrix. They include a multitude of approaches, such as correspondence analysis (CA), nonmetric multidimensional scaling (NMDS), and principal coordinates analysis (PCoA). Their common ground lies in not assuming a specific parametric underlying model for how the data were generated. Different authors group slightly different methods under this label. Warton, Wright, and Wang (2012), for example, exclude CA, while Roberts (2019) explicitly includes it. We follow the wider definition of Roberts (2019) and consider constrained correspondence analysis (CCA) as an example of a distance‐based method. An alternative designation for this group is *algorithmic* or *algorithm‐based* (Warton, Foster, De'ath, Stoklosa, & Dunstan, 2015).

In distance‐based method, the researcher takes the data's statistical properties into account when selecting a distance metric. For instance, Minkowski distances (e.g., Manhattan and Euclidean) assume a constant variance across all mean values (ter Braak & Prentice, 1988) whereas species abundances often show a quadratic mean–variance relationship (Routledge & Swartz, 1991; Yamamura, 1999). Whether a distance metric is appropriate depends on the properties of the data and the aim of the study, as each metric extracts different information from the raw data. The choice is complicated by the vast amount of available metrics (see Legendre & Legendre, 2012). An alternative to distance‐based analyses that accounts for mean–variance relationships and incorporates ecological assumptions is the model‐based approach.

The model‐based approach consists of explicitly specifying a statistical model of the process that generated the observed data (Warton, Foster, et al., 2015). This includes properties such as marginal distributions and corresponding parameters, overdispersion, zero inflation, mean–variance relationship, and correlation structure, all of which can be flexibly tailored to the data and the research question. While this approach is ubiquitous in univariate analyses (Bolker, 2008; Zuur, Ieno, & Elphick, 2010), it has long been uncommon in multivariate ecological analyses, largely due to the absence of suitable models (Anderson, 2001). However, advances in statistical theory and computation power have led to a surge of models for multivariate abundance data. Recent examples include hierarchical modeling of species communities (Ovaskainen et al., 2017), generalized joint attribute modeling (Clark, Nemergut, Seyednasrollah, Turner, & Zhang, 2017), and multivariate generalized linear models (MvGLM, Warton et al., 2012).

In MvGLM, a separate univariate GLM is fit to each taxon, with each model using the same predictors. Univariate GLMs are a flexible method and are strongly advocated for the analysis of count or occurrence data as they can handle different residual distributions and mean–variance relationships (O'Hara & Kotze, 2010; Szöcs & Schäfer, 2015; Warton & Hui, 2011). Extending them to multispecies abundance data was thus a natural starting point for multivariate model‐based analyses (Warton et al., 2012). The univariate models are combined by summing their test statistics, which allows for inference on the whole community. The use of MvGLM, facilitated by an easy‐to‐use implementation in R (in the *mvabund* R package, Wang, Naumann, Eddelbuettel, Wilshire, & Warton, 2019), has steadily increased within the ecological community. However, direct comparisons of MvGLM to other methods remain rare, with a few exceptions. Warton et al. (2012) showed that MvGLMs, in contrast to distance‐based methods, can differentiate between location (difference in mean) and dispersion (difference in mean–variance relationship) effects. Szöcs et al. (2015) found that the statistical power of MvGLMs was higher or at least equal to that of principal response curves (a form of redundancy analysis) when used for the analysis of ecotoxicological semifield studies. However, systematic studies of data sets with known properties are lacking and this paucity of studies hampers our capacity to make informed decisions on the selection of methods for multivariate data analysis.

We compared the performance of MvGLMs to differentiate between causal and noise variables to three methods of data analysis: constrained quadratic ordination (CQO), which is also model‐based, canonical correspondence analysis (CCA), and distance‐based redundancy analysis (dbRDA), which are distance‐based. We applied the methods to 190 combinations of abundance data sets and explanatory variables. The abundance data differed in distributions and sample sizes. Based on the assessment of a variable's statistical significance, false‐positive rate (FPR) and false‐negative rates (FNR) were calculated.

## 2 MATERIALS AND METHODS

### 2.1 Data generation

**Figure 1:**
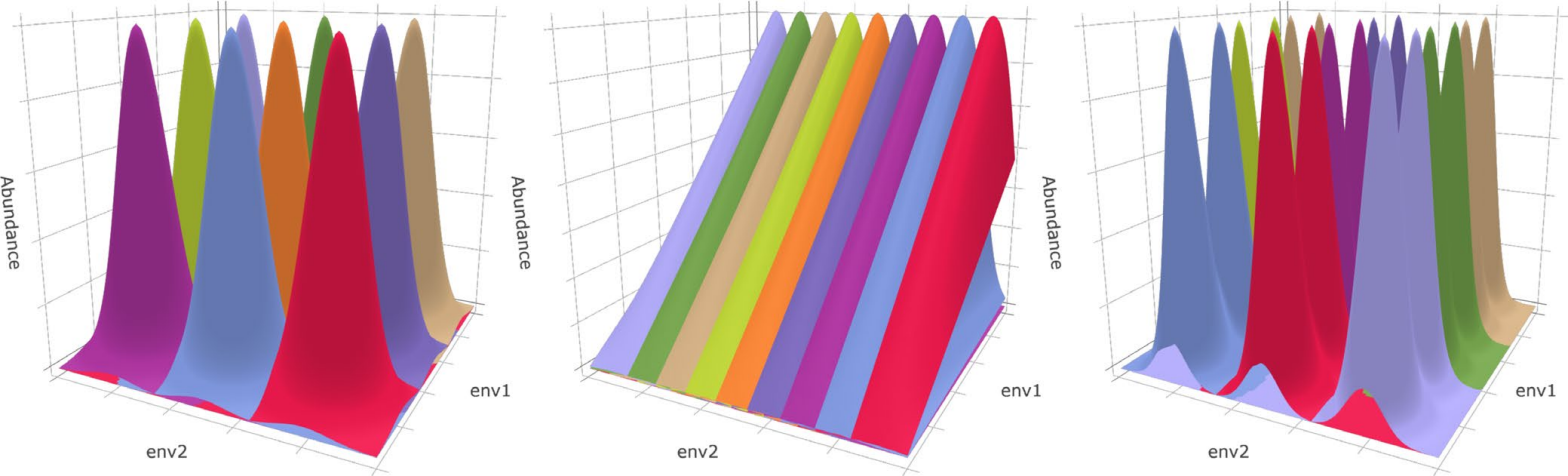
Simulated abundance responses along two causal variables (env1 and env2). Response combinations are as follows: unimodal–unimodal (left), unimodal–linear (middle), and unimodal–bimodal (right). The vertical axis indicates abundance. The different colors represent different species. All the examples show the unsampled abundance matrix **Y_Large_** of type I communities

**Figure 2:**
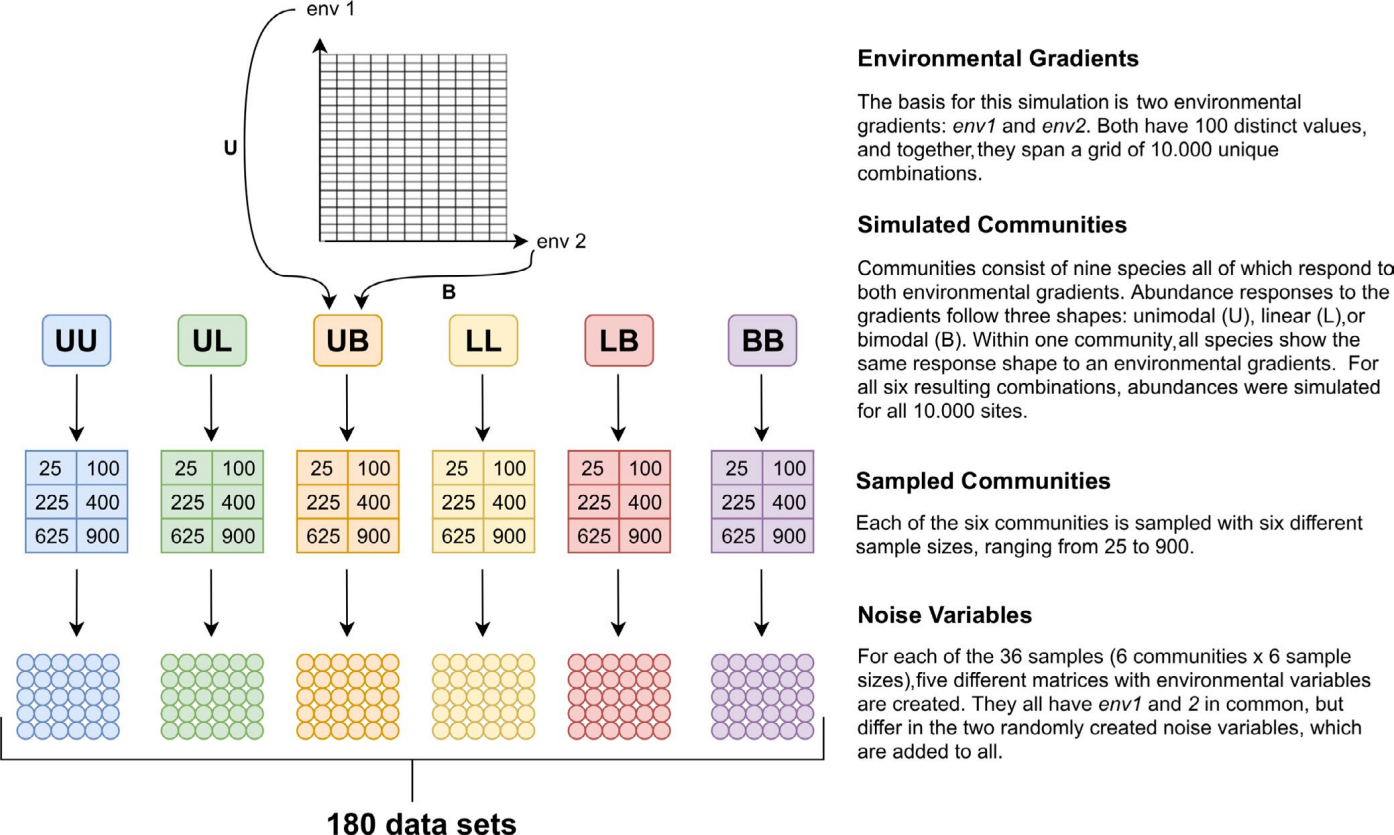
Flowchart of the type I community simulations. The environmental space is comprised out of two variables (env1 and env2) and 10,000 unique sites. At each site, the abundances of nine species are simulated. Abundance responses to environmental gradients display three shapes: unimodal (U), linear (L), and bimodal (B). All nine species of one community show the same response shape (with varying parameters) to one gradient, but response shapes can differ between gradients. All six possible combinations of response shapes are sampled with six different sample sizes spanning from 25 to 900. Before these data are analyzed, two noise variables are added to **X**. For each response shape–sample size combination, five different pairs of noise variables are appended to **X**

Species abundances were simulated as counts, a common abundance measure in ecology (Warton, 2008b). Abundances were stored in **Y**, an *N* × *S* matrix of responses, in this case, the abundances of *S* species, *s* = 1 … *S*, at *N* sites, *n* = 1 … *N*. The species in **Y** responded to environmental variable *x_m_* with one of three different response types: unimodal (*U*), linear (*L*), or bimodal (*B*), as shown in Figure 1. Unimodal responses are most common in nature (Jansen & Oksanen, 2013; Lawesson & Oksanen, 2002) and bimodal shapes are expected to occur when competition restricts realized niches to gradient extremes (Hardin, 1960; Mueller‐Dombois & Ellenberg, 1978). Linear responses may be the result of a stressor gradient shaping communities or may arise if the sampled gradient range is short relative to the species' tolerance. The environmental variables are stored in **X** an *N* × *M* matrix with *M* environmental variables, *m* = 1 … *M*. We simulated three different types of communities. The main focus of this study is the type I communities which are described below. Type II and type III communities represent communities with more heterogeneous responses to environmental variables and may be considered more realistic. They were used to evaluate the robustness of the results and conclusions based on type I communities. They are described in a separate section. The simulation process for type I communities is visualized in Figure 2.

We simulated abundances along two environmental gradients env1 and env2, which henceforth will be referred to as causal variables to differentiate them from the noise variables. Both causal variables consist of the natural numbers from 1 to 100. Each possible combination of the two is a site, that is, the total number of sites *N* = 10,000. **Y_Large_** holds the simulated abundances for all 10,000 sites. This data set is larger than most ecological field data sets, and fitting models to it would have required considerable computation time. Therefore, we sampled from **Y_Large_** with six different sample sizes (25, 100, 225, 400, 625, and 900) to obtain **Y_Sample_**. Depending on the sample size *n*, a set number ($\sqrt{n}$) of sampling locations per causal variable were chosen. These locations always included the variable's minimum and maximum values (i.e., 1 and 100); between those, the locations were equidistantly distributed. The abundances of all species at all combinations of sampling locations constitute **Y_Sample_**. All species show the same response type toward each causal variable, but response types can differ between variables (Figure 1). This setup allows for six communities each with a different combination of response types, including those with identical response types to both variables (Figure 2). The communities are labeled with their abbreviated response types, for example, UB for a community in which species' abundances respond unimodally to the first and bimodally to the second causal variable (Figure 1c).

Unimodal responses were simulated using the Gaussian response model (Gauch & Whittaker, 1972) expanded to multiple dimensions (Equation 1).

(1) $$y_{s,n}=\prod_{m}^{M_{\text{uni}}}C_{s,m}\times \exp\left(-\frac{{\left(x_{m,n}-u_{s,m}\right)}^{2}}{2t_{s,m}^{2}}\right)$$

where *u_s,m_* is the position of the optimum (i.e., the point with the highest abundance) of species *s* along the environmental variable *m*, *t_s,m_* is the tolerance of species *s* toward that variable and determines the width of the unimodal curve, and *c_s,m_* is the maximal abundance of species *s* on environmental variable *m*. *M*_uni_ is the number of unimodal environmental variables. Linear responses were simulated by multiplying the environmental variables with a coefficient *β* (Equation 2).

(2) $$y_{s,n}=\prod_{m}^{M_{\text{lin}}}x_{m,n}\times \beta_{s,m}$$

Bimodal responses were simulated by adding two unimodal models with different optima *u_s,m_*.

This way we obtained *M* = 2 abundance values *y_m,s,n_* per species and site. To obtain a single abundance *y_s,n_* for each species at each site, we multiplied the abundances of each environmental variable. By multiplying instead of adding the abundance values, we ensured that a species is absent from sites where its abundance is zero for one of the gradients, that is, is outside of its niche. The products were rounded down, as abundances can only take integer values.

After the abundances were simulated, noise variables were appended to the matrix of environmental variables **X**. They were simulated from a standard normal distribution, scaled to the same magnitude as the causal variables, and restricted to be orthogonal to them and to each other. We obtained five different versions of these noise variables by altering the random number generation seed, giving us five different versions of **X** per sampled community **Y**_Sampled_. In total, we sampled six different communities six times each and have five matrices with environmental data per sample, resulting in 180 data sets per method of data analysis.

The simulated communities are a simplification of ecological field data. They consist of only nine species and are neither high dimensional nor do they exhibit intercorrelation. However, they are not normally distributed and sparse, thereby featuring two of the common issues mentioned above. This relative simplicity eases interpretation of the results. The simulation process is visualized in Figure 2. More details on the parameterization of the models are provided in Table A1.

### 2.2 Type II and III communities

We simulated two further types of communities to explore the methods' performance when used for more heterogeneous communities. Type II communities consist of only three species. Each species shows the same response shape toward both gradients, but the response shapes differ between species. They contrast with type I communities, where all species exhibit a uniform response shape and allow us to test the influence of deviations from this uniform response on the results. We simulated five type II communities with 625 sites and different random number generation seeds. The parameters were chosen so that the total abundance over all sites was equal for all species (Table A2). Type III communities represent more realistic assemblages. They harbor 30 species sampled at 625 sites, and species abundance distributions (SADs) were simulated with a Gambin model (Ugland et al., 2007) with 10 octaves and a shape parameter of 5 using the gambin R package (Matthews et al., 2014). Octaves followed the common log2 series already used by Preston (1948). The actual maximal abundance was randomly sampled from the interval of the respective octave, and the tolerance was set to the same number as the maximal abundance. All species respond unimodally to both gradients, and the locations of their optima were randomly sampled from all coordinates of the grid. Again, five different communities were simulated. All SADs were drawn from the same Gambin model with different random number generation seeds.

### 2.3 Overview of methods

In the following, the methods of data analysis will be introduced briefly. Each section is concluded with details on how we applied the method in this study.

#### 2.3.1 Multivariate generalized linear models

A MvGLM consists of *S* separately fitted univariate GLMs. The likelihood ratio test statistics of all univariate models (i.e., species) are added for each environmental variable to obtain the sum‐of‐likelihood‐ratio statistics. For these statistics, *p*‐values related the null hypothesis that a given environmental variable has no effect on the mean community abundance can be calculated. We fit MvGLMs with Poisson, negative binomial (both with log‐link), and Gaussian residual distributions (with identity link) to each community and compared their Dunn–Smyth residual plots (Dunn & Smyth, 1996) and Akaike's information criteria (AIC, Akaike, 1974). We did not test models with quadratic or higher order polynomial terms. The likelihood ratio test statistic was calculated for the best fitting model (least patterns in residuals and lowest AIC). To estimate *p*‐values, we used a residual permutation bootstrap with 1,000 repetitions (Davidson & Hinkley, 1997).

#### 2.3.2 Constrained quadratic ordination

Like the MvGLM, the CQO is related to the GLM. It is based on vector generalized linear models (VGLMs), which are a further generalization of GLMs. All GLMs are special instances of VGLMs, just like linear regression is a special instance of a GLM. They are not restricted to the exponential family, include multivariate response models, and can explicitly model other response parameters than the mean (e.g., the variance or higher order moments). CQO builds on reduced‐rank VGLMs, in which the *M* original predictors are reduced to *R* latent variables ν. This entails the reduction of the hat matrix **H**, which holds the regression coefficients *β*, to a rank *R* matrix **H_R_**. So unlike a MvGLM, CQO reduces the data's dimensionality, and in contrast to most ordination techniques (including dbRDA and CCA), the researcher specifies the number of latent variables (i.e., dimensions) a priori. **H_R_** is decomposed into two matrices $H_{R}^{T}={\mathrm{AC}}^{T}$, where $H_{R}^{T}$ denotes the transpose of **H_R_**. The latent variables ν are the linear combinations of the constrained coefficients **C** *^T^* and the sites‐by‐predictor matrix **X**. This means that the higher the constrained coefficient of a given predictor is, the more the influence it has on the corresponding latent variable. **A** holds the regression coefficient of the latent variables. CQO extends this model by adding a quadratic term (cf. Equation 3).

(3) $$\eta_{s}=\beta_{\left(s\right)1}+\beta_{\left(s\right)2}\nu +\beta_{\left(s\right)3}\nu^{2}$$

*β*_1_ is the intercept term, and η is the linear predictor. It assumes symmetric and unimodal responses to the latent variables. CQOs were run with Poisson residual distribution and the canonical log‐link function. The four explanatory variables were scaled and centered before fitting the models. The effective nonlinear degrees of freedom were set to 1.5 as suggested by Yee (2015). Each model was run fifty times, and the deviances of each run were compared. If the lowest deviances are too far apart, the solution might be local and the model should be refitted. Here, we fit the model again, until the difference between the lowest and the fifth lowest deviance no longer exceeded 3. In its current implementation in the VGAM R package (Yee, 2019), CQO does not provide *p*‐values (but see Yee, 2010). To compare its results with the other methods, we calculated pseudo‐*p*‐values for the CQO (details of the procedures can be found in the Appendix 1). Shortly, to determine the pseudo‐*p*‐value of environmental variable *m*, we permuted the variable 100 times and fit a CQO to each permuted data set. For every model, the absolute values of the constraint coefficient across both latent variables were added for environmental variable *m*, to obtain the test statistic $\sum C_{\nu_{X_{m}}}$. The proportion of test statistics of permuted data sets that were larger than that of the unpermuted data set is the pseudo‐*p*‐value. All models were fit with ranks 1 and 2. The optimal number of ranks was found to be 2 for all models, determined by the AIC as proposed by Yee and Hastie (2003).

#### 2.3.3 Canonical correspondence analysis

Canonical correspondence analysis is the heuristic solution to restricted Gaussian regression (Zuur, Ieno, & Smith, 2007). In the latter, one tries to estimate the parameters *u*, *t*, and *c* of a Gaussian response model (see Equation 1), but instead of the measured environmental variables, their linear combinations are used as ***x***. Though it is possible to estimate the parameters with iteratively reweighted least squares in a GLM, this was to computationally intensive at the time the method was proposed by Gauch and Whittaker (1972). Instead, ter Braak (1986) proposed to approximate the results by CCA, which is valid as long as: All species have equal tolerances *t* and maximal abundances *c*, their responses are unimodal and symmetrically bell‐shaped, and their optima *c* are spread uniformly in the ordination space. These assumptions are collectively known as the *species packing model*. Palmer (1993), Johnson and Altman (1999) and Zuur (1999) confirmed the validity of the approximation and its robustness toward violations against the species packing model in simulation studies. Today, CCA is one of the most widely used and cited multivariate statistical methods in ecology (ter Braak, 2014).

An iterative algorithm is used to obtain estimates. First, arbitrary values are assigned to the site scores (positions of sites in latent variable space, **Z**). These are used to calculate the species optima *u* (henceforth species scores) as in Equation 4

(4) $$u=D_{c}Y^{t}Z$$

where **u **= (*u*_1_ … *u_S_*)*^t^*, **D** *_c_* is a diagonal matrix with the abundance of species *s* across all sites as its *s,s*‐th element, and **Y** ^t^ denotes the transpose of **Y**. The species scores are in turn used to calculate the site scores as their weighted average **Z**_wa_ (Equation 5)

(5) $$Z_{\text{wa}}=D_{r}^{-1}\mathrm{Yu}$$

where **D**_r_ is a diagonal matrix with the abundance of all species at site *n* as its *n,n*‐th element, and $D_{r}^{-1}$ denotes the inverse of **D**_r_. **Z**_wa_ is regressed against **X** to obtain the weighted regression coefficient *α*.

(6) $$\alpha ={\left(X^{t}D_{r}X\right)}^{-1}X^{t}D_{r}Z_{\text{wa}}$$

Lastly, **Z** is calculated as the product of **X** and **α**. This procedure is repeated until convergence.

The distance between sites (scaling 1) or species (scaling 2) in a CCA approximates their two‐dimensional chi‐square distance, that is, the Euclidean distance between the expected abundances under the null hypothesis, that abundances do not change along environmental variables and the actual data. Explanatory variables were scaled and centered. Hypothesis tests for environmental variables can be conducted using a pseudo‐*F*‐statistic with permuted residuals (Legendre, Oksanen, & Braak, 2011) and the null hypotheses that the effect of the variable on the response is equal to zero after accounting for the effect of all other variables. Hypothesis tests were conducted with 999 permutations.

For type I and II communities, we did not transform the abundances as all species had similar or equal maximal abundances. For type III communities, the CCA was run with untransformed, square root‐transformed, base 2 log‐transformed, and Hellinger‐transformed abundance data.

#### 2.3.4 Distance‐based redundancy analysis

Distance‐based redundancy analysis (dbRDA) is a variation of the commonly used redundancy analysis, proposed by Legendre and Anderson (1999). It is not based on one specific distance measure but instead can adopt any chosen measure. It is the constrained form of principal coordinate analysis (PCoA, Legendre & Anderson, 1999), which will be shortly addressed here. In PCoA, **Y** is transformed into a centered distance matrix Δ. The columns of the matrix **PC** are the eigenvectors of Δ scaled to a length that is equal to the square root of their eigenvalues (Gower, 1966). Each row of PC gives the eponymous *Principal Coordinates* of one observation. In a dbRDA, this matrix **PC** is linearly related to the explanatory variables by an RDA. The dbRDA preserves the distance metric of Δ, which can be metric, semi‐, or nonmetric. dbRDA was highlighted by Szöcs et al. (2015), because the possibility to use asymmetrical distance metrics makes them appealing for sparse data sets. We used the Bray–Curtis distance, which is the reciprocal of the Steinhaus coefficient (Motyka, 1947), to calculate Δ. As in CQO and CCA, environmental variables were scaled and centered. The significance tests for explanatory variables are calculated using a pseudo‐*F*‐statistic in the same manner as for the CCA. dbRDA of type III communities was run with untransformed, square root‐transformed, base 2 log‐transformed, and Hellinger‐transformed abundance data.

### 2.4 Comparison of methods

The benefit of using simulated rather than field data are twofold: (a) There is a clear dichotomy between causal and noise variables, and (b) we know whether a given explanatory variable is causal or noise. This enables us to compare the methods in terms of their classification error rates. To this end, we calculated false‐positive (FPR) and false‐negative rates (FNR) for each method.

(7) $$\text{FPR}=\text{FP}/\left(\text{TN}+\text{FP}\right)$$

(8) $$\text{FNR}=\text{FN}/\left(\text{TP}+\text{FN}\right)$$

where FP is a false positive, TN a true negative, FN a false negative, and TP a true positive. A false positive occurs when a noise variable is classified as causal, whereas a false negative when a causal variable is classified as noncausal. True positives and negatives are instances where the variable is labeled correctly. An FPR of 0.5, for example, would indicate that half of all variables that were determined to be causal are in fact noise. Variables with a *p*‐value lower than the significance level (*α*) were classified as causal whereas all variables with *p* > *α* were classified as noise. To alleviate the problematic dichotomy of statistical significance (Greenland et al., 2016), we use five different significance levels *α* (0.01, 0.03, 0.05, 0.07, and 0.1). This allows us to evaluate trends in classification strength over different thresholds.

### 2.5 Software

We used R 3.4.4 (R Core Team, 2018) for all simulations and analyses. MvGLM was conducted with mvabund 3.13.1. (Wang et al., 2019), dbRDA and CCA with vegan 2.5–2 (Oksanen et al., 2018), and CQO with VGAM 1.0–5 (Yee, 2019). All calculations were conducted on an Ubuntu 18.04 machine with 64‐bit, 8 GB RAM, and 1.6 GHz.

## 3 RESULTS

We report the means and standard deviations of *p*‐values of MvGLM, CQO, CCA, and dbRDA for all explanatory variables on type I communities (Table 1); *p*‐values for all combinations of response shapes and sample sizes as well as type II and III communities are given in Tables A3, A4, A5, A6, A7, A8.

**Table 1:** Mean *p*‐values ± standard deviations of the causal (env1 and env2) and noise variables from multivariate generalized linear models (MvGLMs), constrained quadratic ordination (CQO), canonical correspondence analysis (CCA), and distance‐based redundancy analysis (dbRDA) on type I communities

	MvGLM	CQO	CCA	dbRDA
env1	0.006 ± 0.0275	0.067 ± 0.127	0.264 ± 0.433	0.002 ± 0.007
env2	0.009 ± 0.0311	0.090 ± 0.190	0.264 ± 0.433	0.003 ± 0.001
Noise	0.650 ± 0.280	0.680 ± 0.268	0.399 ± 0.348	0.450 ± 0.277

**Figure A1:**
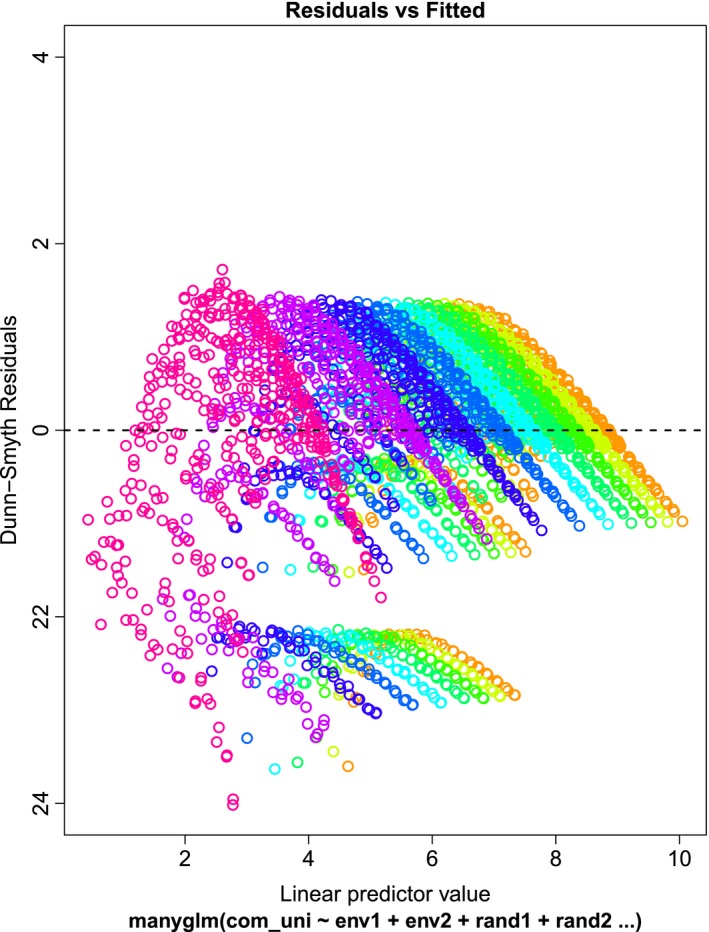
The Dunn–Smyth residuals of the LL community sampled with 400 samples plotted against the linear predictor. A pronounced arched pattern can be observed for every single species (different colors)

In most MvGLMs, negative binomial residual distribution achieved the lowest AIC and the best fit to model assumptions. The plot of Dunn–Smyth residuals against the linear predictor of LL (Figure A1) showed arched patterns, which could indicate that the residuals were not independent of the explanatory variables. Nevertheless, we used a negative binomial residual distribution because the visual inspection of the QQ plots suggested that it resulted in a better fit than Poisson or Gaussian distributions.

MvGLMs' *p*‐values for both causal variables and all response type combinations were low (Table 1 and Figure 4). The *p*‐values of the linear variable in LB and UL and of the bimodal variable in UB were higher at the smallest sample size than at higher ones (Table A3). Otherwise, the sample size had no effect on the *p*‐values of the causal variables, which were often minimal (1 divided by the number of permutations + 1). The *p*‐values for noise variables were higher and varied strongly. They only fell below the nominal significance level of 0.05 in three models. All three models had the response combination LL, and the low *p*‐values occurred at the sample sizes 225, 625, and 900 (Table A3).

**Figure 3:**
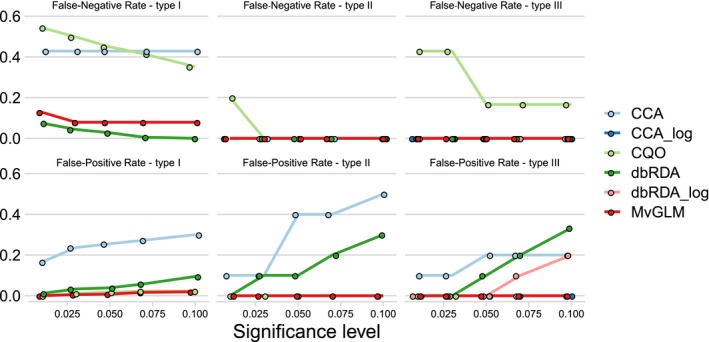
False‐positive rate and false‐negative rate of the four statistical methods canonical correspondence analysis (CCA), constrained quadratic ordination (CQO), distance‐based redundancy analysis (dbRDA), and multivariate generalized linear model (MvGLM) with type I, II, and III communities. CCA_log and dbRDA_log show the results of CCA and dbRDA on base 2 log‐transformed data, which yielded to lower or equal FPR than the other two transformations (see Figure A6). Points are jittered slightly along the *x*‐axis

The FPR was the lowest of all methods (0.008 at *α* = 0.05) and always well below the respective significance level. Overall, FPRs and FNRs of MvGLMs were very low (Figure 3). Interestingly, the *p*‐values of noise variables did not show a monotonic positive relationship with sample size, as we expected. Rather, the response seemed unimodal in UU, LL, and BB, slightly negative in LB and UL, and positive for UB (Table A3).

MvGLM had a FNR and FPR of zero with both type II and type III communities at all significance levels (Figure 3).

**Figure 4:**
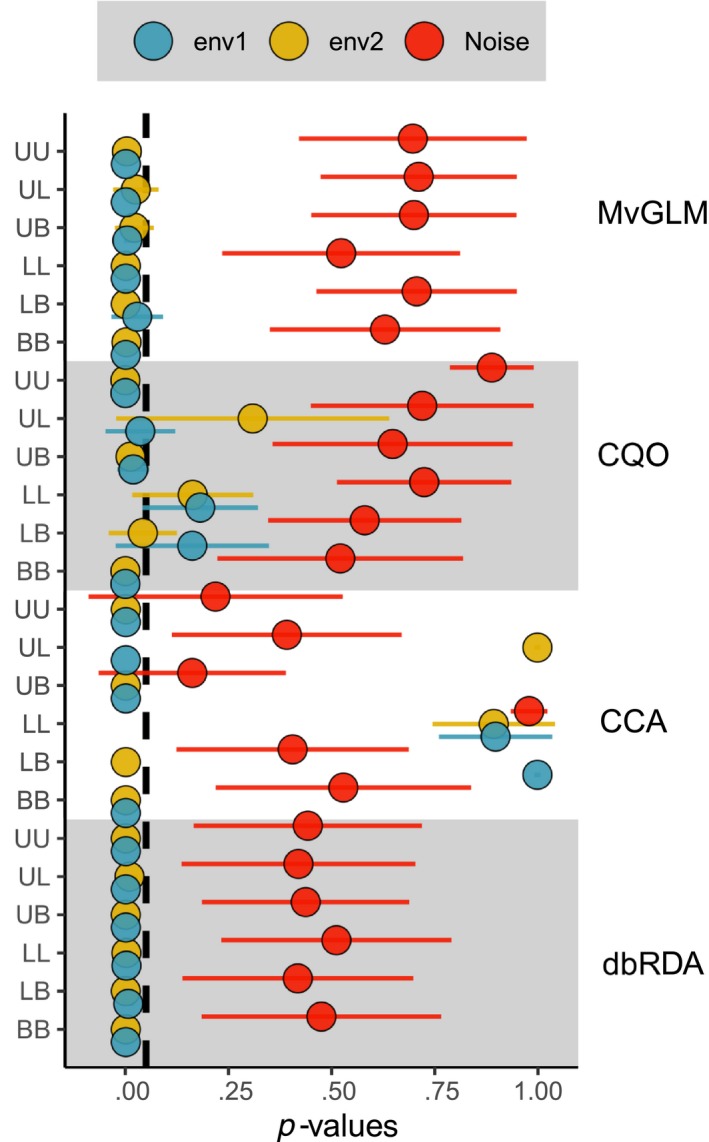
Mean *p*‐values of response combinations (indicated by first letter of response types: unimodal (U), linear (L), and bimodal (B)) for multivariate generalized linear models (MvGLM), constrained quadratic ordination (CQO), canonical correspondence analysis (CCA), and distance‐based redundancy analysis (dbRDA). Blue points are env1, yellow points are env2, and red points are noise variables. Bars show one standard deviation. The vertical dashed line indicates a *p*‐value of 0.05. Only type I communities are shown

Constrained quadratic ordinations' performance strongly depended on the response shape (Figure 4). It failed to converge for UB with sample size 25 and performed best for UU and BB; both had a FNR of 0 FPRs below the average (0 and 0.06, respectively). UB performed slightly worse than UU and BB with an FNR of 0.1 and an FPR of 0.02. As was expected, CQO often assigned high *p*‐values to linear causal variables (Figure 4). The mean *p*‐value of linear variables was 0.15, and their FNR was 0.53. Both unimodal and bimodal causal variables received higher *p*‐values when the other causal variable was linear (Table A4). The mean *p*‐value of unimodal variables excluding those from UL is 0.006 ± 0.022 compared to 0.036 ± 0.084 for the unimodal variable in UL. Similarly, the mean *p*‐value of bimodal variables except for those form LB is 0.004 ± 0.015, and for the bimodal variable in LB, it is 0.042 ± 0.083. This mixed performance leads to relatively high mean *p*‐values for the causal variables (Table 1) and accordingly high FNR and FPR (Figure 3).

Constrained quadratic ordination is the only method that has nonzero FNR in type II and III communities. In type II communities, the FNR is 0.2 for *α* = 0.1 and zero for all other significance levels. In type III communities, the FNR is 0.43 for an *α* between 0.01 and 0.03, and then decreases to 0.17 for all higher significance levels (Figure 3).

Canonical correspondence analysis has the highest mean *p*‐values for causal variables and the lowest for noise ones. Accordingly, the FPR was the highest of all methods (Figure 3). Irrespective of significance level, it is more than one order of magnitude higher than for all other methods. These problems are due to two factors: (a) high *p*‐values for causal linear variables and (b) low *p*‐values for noise variables. The mean *p*‐value for causal linear variables is 0.963 ± 0.094. Additionally, CCAs of LL with sample sizes 400–900 produced constrained inertias (explained variance) of 0 and were therefore excluded from significance testing. Noise variable *p*‐values were especially low in UU and UB (Figure 4), which is interesting since these data sets matched closest with the assumed *species packing model*. In BB, they were markedly higher (Table A5). The impact of different sample sizes was negligible in all response combinations (Table A5).

The FNR was 0 in all type II and type III communities, whereas the FPR depended on the type of transformation. With Hellinger‐, square root and base 2 log‐transformed data, FPR was zero for type II and III communities at all significance levels, except for square root‐transformed data it is at the significance level 0.1 in type III communities where it increased to 0.1. The FPR on untransformed data was higher. It was overall highest, in type II communities with 0.5 at the significance level 0.1. In type III communities, CCA maintained an FPR of 0.1 between the significance levels of 0.01 and 0.03 and increased to 0.2 for higher significance levels.

The dbRDA assigned low *p*‐values to most causal variables (Figure 4). The only *p*‐values of causal variables above the nominal significance level of 0.05 were those of linear variables at a sample size of 25 (Table A6). However, they were below 0.1, so that the dbRDA had an FNR of 0 at *α* = 0.1 Indeed, the FNR was the lowest of all methods (Figure 3). The FPR was relatively high, with 0.039 at *α* = 0.05. *p*‐values were relatively similar for all sample sizes (Table A6).

**Figure A3:**
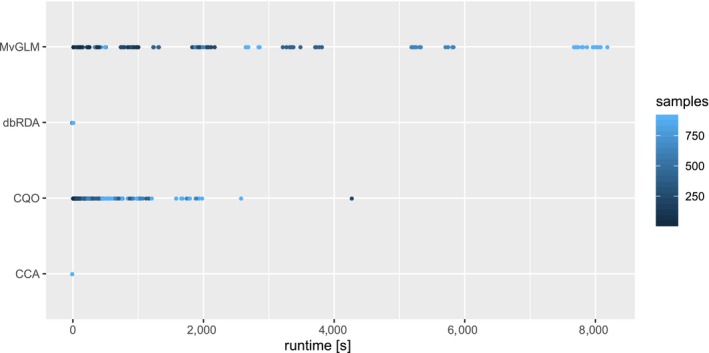
Run times of multivariate generalized linear models (MvGLM), distance‐based redundancy analysis (dbRDA), constrained quadratic ordination (CQO), and canonical correspondence analysis (CCA). The *x*‐axis is scaled with a decimal logarithm. Colors indicate sample sizes

At most significance levels, the FPR of dbRDA in type II and III communities was higher than in type I communities. In type II communities, it was 0.1 at *α* = 0.05 and increased to 0.3 at *α* = 0.1. For type III communities, FPR increased steadily with significance level, reaching 0.3 with Hellinger transformation and 0.33 on untransformed data at *α* = 0.1. Both square root and base 2 log transformation had lower FPRs, reaching 0.2 at *α* = 0.1. Log‐transformed data maintained an FPR of 0 for all sigificance levels below 0.07. Both distance‐based methods were considerably faster than the model‐based ones (Figure A3).

## 4 DISCUSSION

We analyzed 190 simulated abundance data sets that differed in response types and sample sizes with four different statistical methods, to assess the methods' performance when used to differentiate between causal and noise variables. MvGLM and dbRDA performed best with type I communities showing low FPRs and FNRs for all response combinations and sample sizes. CQO assigned high *p*‐values to noise variables, resulting in FPRs lower than those of dbRDA but higher than MvGLMs. However, it had the highest FNR for the lower three significance levels, resulting largely from the high *p*‐values of linear variables. CCA assigned high *p*‐values to linear variables and additionally assigned low *p*‐values to noise variables.

The method performed worst with type I communities, showing the highest FPR at all significance levels and the highest FNR at the two highest significance levels. However, its performance on Hellinger and base 2 log‐transformed data for type II and III communities was as good as that of MvGLM.

MvGLMs had the lowest FPR of all methods and showed the best performance when all community types are considered. The three noise variable *p*‐values that fell below 0.05 all occurred in LL models, which violated the assumption of random residuals and thus would likely be identified as unreliable models. The FNR was also low and all false negatives occurred in communities with the smallest sample size. A drawback of MvGLMs is the long run time due to resampling.

The resampling is used for inference, and since whole sampling units (rows) are resampled, correlation structures between species are preserved (Wang, Naumann, Wright, & Warton, 2012).

Models that explicitly consider correlation structure avoid resampling and can reduce computation time. Such models have been proposed, for example, by Jamil, Ozinga, Kleyer, and Braak (2012) who used the site effect of a generalized linear mixed model to induce equal correlation between all species pairs. A clear drawback of this method is, however, that equal correlation between all species is as (im)plausible as no correlation. Structuring the residual covariance matrix is important as the number of parameters that need to be estimated rises quickly (e.g., 55 in the covariance matrix for 10 species). MvGLMs can use an unstructured correlation matrix, but this is only advisable for data sets with many more sites than species and is computationally expensive. Another option is shrinking the correlation matrix toward identity using ridge regularization (Warton, 2008a, 2011). Both alternatives use generalized estimation equations (GEE) with the sandwich‐type‐estimator of Warton (2011). As GEEs do not provide likelihoods, other test statistics than the likelihood ratio have to be used. Current options are the score and the Wald statistic. However, these methods also require resampling, as asymptotic marginal distributions of regression parameters for GEEs are not specified for data sets with more species than sites. Testing these methods on data sets with known correlation structures could highlight stronger performance differences, as the other methods lack adjustments to these properties.

MvGLMs are the only method considered here that does not provide an easy to use and to interpret method for visualizing the data.

dbRDA was least influenced by different response types and sample sizes in type I communities.

In type II and III communities, however, FPR was higher than that of both model‐based methods. Square root and log transformation lowered FPR compared to dbRDA on untransformed data, but remarkably Hellinger transformation did not lead to a lower FPR. For *α* = 0.75, the FPR of dbRDA on Hellinger‐transformed data was even higher than that of dbRDA on untransformed data.

Small *p*‐values were scarce for noise variables but occurred at all sample sizes and response types. dbRDA's good performance on type I communities is in concert with other simulation studies (Roberts, 2009). These results are only valid for the Bray–Curtis distance metric, which was used here.

Indeed, Yamamura, Blanchet, and Higa (2019) recently demonstrated performance differences between dbRDAs with different distance metrics.

The selection of an appropriate metric is thus a crucial step in any dbRDA analysis. Having to choose a single metric can be avoided by using consensus RDA (Blanchet, Legendre, Bergeron, & He, 2014). In this method, multiple dbRDAs are run, only differing in their distance metric. Site scores on statistically significant axes are combined into one matrix, which acts as a response matrix in a new RDA. This method extracts the information that is common to all individual dbRDAs. Simulation studies comparing properties of consensus RDA with those of individual dbRDA and other methods, distance‐ or model‐based, are lacking. Another avenue for the future development of distance‐based algorithms, in general, would be novel distance metrics, but their development is pending (M. J. Anderson, pers. comm.).

The CCA performed worst on the type I communities of the methods tested and assigned high *p*‐values to all linear variables. As CCA assumes unimodal gradients, which are more frequent than linear ones in nature (Oksanen & Minchin, 2002), this was expected. This study confirmed that CCA should be avoided if exploratory analyses indicate linear relationships, which can occur if the sampled range of a gradient is short relative to the species' tolerance. Noise *p*‐values were lower than in other methods. Most of the low *p*‐values for noise variables occurred in communities with uni‐ or bimodal responses. This is surprising, given that UU fits the expectations of the species packing model perfectly and bimodal models deviate only slightly.

In type III communities, CCA on base 2 log‐transformed data performed as well as the model‐based methods, while the FPR of untransformed data was slightly lower than in type I communities. The latter is likely due to chance; FPR and FNR for both type II and III communities were only based on five repetitions instead of 180 for type I communities, which were the main focus of this study. Overall, the result corroborates earlier findings that CCA is robust against two specific violations of the species packing model: unequal maximal abundances and nonregular distribution of optima in the ordination space.

Newer approaches to CCA that can correct for zero inflation (Zhang & Thas, 2012) or nonlinear relationships between predictor and response variable (Makarenkov & Legendre, 2002) are available but not widely used. Indeed, all of the methods we tested here can include quadratic terms which would most likely have resulted in better fitting models for unimodal and bimodal predictors. Their application is uncommon in CCA and RDA and could be the scope of future studies.

Similar to the CCA, CQO assigned high *p*‐values to linear variables. It also assumes unimodal responses, and the nondetection of causal linear gradients was expected. The *p*‐values for linear variables of CQO were markedly lower than in the CCA; however, the *p*‐value of the second variable in these models tends to increase. Overall, this resulted in a high FNR. The FPR was still lower than for both distance‐based methods but slightly higher than for MvGLM. These results reflect the performance of CQO when combined with our novel approach to compute *p*‐values. CQO has only rarely been used in ecological studies and mostly within fisheries research (Carosi, Ghetti, Porta, & Lorenzoni, 2017; Top, Tarkan, Vilizzi, & KarakuÅŸ, 2016; Vilizzi, Stakenas, & Copp, 2012). ter Braak and Šmilauer (2015) suggest that this is due to limitations on the number of species that can be included, a steep learning curve, and numerical instability. This study confirmed that in its current state, the method has issues with linear response types but can handle alteration of the symmetrical unimodal bell shape.

Constrained Quadratic Ordination encompasses many options that we did not test. They include different models for the tolerance matrix, further marginal distributions, and additive models. Considering all plausible combinations of these exceeded the scope of this study, but could improve performance. We refer the interested reader to the comprehensive treatment in Yee (2015).

Our findings suggest that MvGLMs can be applied in a wide variety of settings. None of the data sets or their respective properties resulted in high FPR or FNR. CQO had low FPR rates in all tests but had the highest FNR. However, as stated before, many options of CQO remained unexplored in our study, which might remedy the problems. In type I communities, CCA had high FNR with linear responses and a high FPR with unimodal responses. We thus caution against the use of CCA if exploratory analysis indicates linear relationships. Lastly, dbRDAs performed well with type I communities, but worse with type II and III communities. Data sets with a high number of species or stronger abundance differences might pose problems to dbRDA that can only partially be alleviated by transforming the data.

Our study is the first to directly compare the methods. Warton et al. (2012) compared MvGLMs to CCA and RDA (not dbRDA). They showed that only MvGLMs successfully differentiate between the location effect (difference in means) and dispersion effect (difference in variance).

Yamamura et al. (2019) compared a Bayesian hierarchical model (BHM) with dbRDA focusing on the impact of incomplete and unequal sampling. They found that the BHM and dbRDA estimated the dependence of the species abundances on environmental variables similarly well. However, the BHM differs strongly from MvGLM and CQO. Roberts (2019) compared different distance‐based and model‐based ordinations compared with our study. His results contrasted with ours in that the distance‐based methods (NMDS and t‐distributed stochastic neighbor embedding) outperformed model‐based methods (Bayesian ordination and regression analysis, and random effects ordination), when used to determine environmental drivers of community composition.

Comparative studies of multivariate methods, in general, are common. Especially, ordination techniques such as CCA and RDA were subject to extensive testing in the 1970s and 1980s (Gauch & Whittaker, 1972; Gauch, Whittaker, & Wentworth, 1977; Kenkel & Orloci, 1986). Roberts (2008) and Roberts (2009) compared dbRDA, CCA, and multidimensional fuzzy set ordinations. Roberts (2008) used simulated data sets to this end, whereas Roberts (2009) used four different field data sets. Both studies concluded that dbRDA outperforms CCA, which we also find for type I community data but not for type II or type III. CQO is occasionally tested in comparisons of individual and community‐level species distribution models (Baselga & Araújo, 2009; Maguire et al., 2016), where they are an instance of the latter. Generally, they exhibited a similar performance as classical models (e.g., GLMs or Regression Trees).

Future studies could improve the realism of the simulated communities by using more complex response patterns like beta‐functions (Austin, Nicholls, Doherty, & Meyers, 1994), which add asymmetries to bell‐shaped curves. However, in a study of Oksanen and Minchin (2002) only about 20% of the responses were strongly skewed, whereas symmetric and bell‐shaped responses were most common. Alternatively, asymmetry could be introduced through random terms added to abundances, environmental variables, or both (McCune, 1997). When correlated random terms are added to both, this would engender endogeneity (a nonzero covariance between the residuals and one or more explanatory variables). Simulations with induced endogeneity would be interesting as this phenomenon is underappreciated by ecologists (Armsworth, Gaston, Hanley, & Ruffell, 2009; Fox, Negrete‐Yankelevich, & Sosa, 2015). Observation and measurement are sources of errors in field data sets, and both can be represented in a model via binomial functions as in N‐mixture models (Royle, 2004). This would be interesting to examine the effects of regression dilution (Frost & Thompson, 2000; McInerny & Purves, 2011).

It would also be of great interest to compare the methods' performance with presence–absence data instead of abundance data as novel options for analysis have recently emerged for this less informative but more available type of data (Podani, Pavoine, & Ricotta, 2018; Sander, Wootton, & Allesina, 2017; Tovo et al., 2019).

Our study shows that model‐based multivariate inference can outperform more frequently used distance‐based methods. The answer to our eponymous question is thus: Not categorically, decisions should be made on a case‐by‐case basis. As model‐based methods are still at an early stage, new developments and increases in computation speed can be expected. An especially active area of development is models using joint probability distributions (Clark, Gelfand, Woodall, & Zhu, 2014; Pollock et al., 2014) that estimate the joint distribution of all species conditional on the environmental variables instead of only using the marginal distribution of every species' abundance. A common interest of many joint models is to infer biotic interactions from the residuals of the species–environment interaction, as these two sets of predictors (biotic and abiotic) were shown to have little redundancy (Meier et al., 2010). Some of the models also anticipate the growing challenges of Big Data for ecology (Hampton et al., 2013). Generalized linear latent variable models, for example, include latent variables instead of random effects to capture residual correlation, which considerably reduces the size of the variance—covariance matrix (Niku, Warton, Hui, & Taskinen, 2017; Warton, Blanchet, et al., 2015). In hierarchical modeling of species communities (Ovaskainen et al., 2017), this approach is coupled with a fourth corner model (including species traits, Legendre, Galzin, & Harmelin‐Vivien, 1997) and phylogenetic relationships to create a flexible and comprehensive framework for community data analysis. In a similar vein, generalized joint attribute models allow for different kinds of data (e.g., continuous, discrete counts, ordinal counts, and occurrence) to be included in the same response variable and have outperformed Poisson GLM on discrete count data and a Bernoulli GLM on binary host status data in a recent simulation study (Clark et al., 2017).

Another recent and promising development in ecology is copula models (Anderson, Valpine, Punnett, & Miller, 2019; Popovic, Hui, & Warton, 2018; Popovic, Warton, Thomson, Hui, & Moles, 2019).

Anderson et al. (2019) highlighted a combination of the model‐ and distance‐based approaches. They proposed a copula model of ecological count data (see Hofert, Kojadinovic, Mächler, & Yan, 2018, for an introduction to copula models), which consists of (a) fitting a copula model to the data, (b) simulating new count data with this copula, and (c) visualizing the centroids of the actual data and of the simulated data sets in a metric multidimensional scaling. In light of the good performance of dbRDA in our study, this proposal, to join features from both approaches, should be further pursued. It is now essential that ways to infer ecological processes from the modeled patterns develop at a similar pace as these models, to avoid confusing statistical artifacts with genuine biological signals (Dormann et al., 2018).

## CONFLICT OF INTEREST

None declared.

## AUTHOR CONTRIBUTIONS

JFJ and RBS conceived the experiment. JFJ conducted the simulation and the analyses. JFJ and RBS wrote the manuscript.

## Figures and Tables

**Figure A2:**
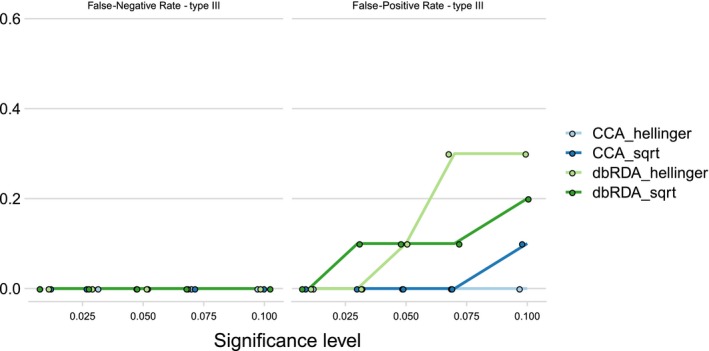
False‐positive and false‐negative rates of canonical correspondence analysis (CCA) and distance‐based redundancy analysis (dbRDA) on square root‐ and Hellinger‐transformed abundance data. Points are jittered slightly along the *x*‐axis

## Data Availability

All data as well as R scripts are available in the associated GitHub repository: https://github.com/JonJup/Should-ecologists-prefer-model-over-distance-based-multivariate-methods

## APPENDIX 1

### Calculating pseudo‐*p*‐values for CQO

Currently, the VGAM R package (Version 1.1‐1, Yee, 2019) does not implement hypothesis tests regarding the predictors in a CQO. As we relied on *p*‐values to compare the tested methods, we calculated pseudo‐*p*‐values for CQO using a permutation‐based test. We used the absolute sum of constrained coefficients (*C*_Σ_) as the test statistic. The constrained coefficient *C_ij_* is the weight of the variable *X_i_* on the latent variable *v_j_*; the higher the *C_ij_* is, the stronger the *X_i_* influences *v_j_*. By summing *C_i_* over all latent variables, we test the impact that *X_i_* has on the model as a whole. In this summation, we used the absolute values and removed the mathematical sign as these only signify the direction of influence, not its magnitude. We do not know what distribution to expect from this statistic or if it adheres to a specific distribution. The method of choice for such cases is permutation‐based tests, which produce pseudo‐*p*‐values (Legendre & Legendre, 2012). Their general approach is as follows: A test statistic *T* is computed for the data set of interest *D*, with X,Y∈D. Some property of *D* (e.g., the rows of *X* or *Y*) is permuted *n* times, and the same test statistic is calculated for each of the permuted data sets *D* ^*^. The pseudo‐*p*‐value can then be calculated as follows:

$$p=\frac{\sum_{j=1}^{n}\left(k_{j}\right)}{n+1}\;\text{with}\;k_{j}=\left\{\begin{array}{cc} 1 & \text{if}\;T_{j}^{*}\geq T\\ 0 & \text{else} \end{array}\right..$$

We permuted the predictors. Each predictor was tested separately so that in any one model only one predictor was permuted while the other remained in their original order.

## APPENDIX 2

### Appendix figures and tables

Table A1 shows the model parameters used in the simulations of type I communities. The optimum parameter *u* is the only instance of a parameter that is relevant to both gradients and differs between them. Table A2 shows the same information for type II communities.

Figure A1 shows the arched patterns in Dunn‐Smyth residuals referred to in the result section

Figure A2 displays false positive and negative rates for dbRDA and CCA on square root and Hellinger transformed data

Figure A3 shows the runtimes of the four methods

Tables A3, A4, A5, A6 show the *p*‐values of all explanatory variables (env1, env2 and noise) for all response combinations and methods in type I communities.

Tables A7 and A8 show the same information for type II and III communities.

**Table A1:** Model parameters used for unimodal (*U*), linear (*L*), and bimodal (*B*) responses in simulations of type I communities

	*c*	*t*	*u*	*β*
UU	100	7.5	20, 50, 80	x
UL	100	7.5	10, 20, 30, 40, 50,60,70,80,90	0.1
UB	100	5	20, 50, 80, [10, 30], [40, 60], [70, 90]	x
LL	x	x	x	0.1, 0.2125, 0.3250, 0.4375, 0.5500, 0.6625, 0.7750, 0.8875, 1.0000
LB	100	6	[5, 25], [25, 45], [35, 55], [55, 75], [75, 95]	0.1
BB	100	6	[5, 25], [35, 55], [75, 95]	x

Note

An x indicates that the parameter is not relevant to the respective gradient type. *c* is the maximal abundance, *t* the tolerance, *u* the location of the optimum, and *β* the linear response parameter. Values in square brackets are the pairs of optima for bimodal gradients.

**Table A2:** Model parameters used in simulations of unimodal (U), linear (L), and bimodal (B) responses in type II communities

	*c*	*t*	*u*	*β*
*U*	10	7.5	50	x
*L*	x	x	x	0.037
*B*	5	7.5	[25, 75]	x

Note

An x indicates that the parameter is not relevant to the respective gradient type. *c* is the maximal abundance, *t* the tolerance, *u* the location of the optimum, and *β* the linear response parameter. Values in square brackets are the pairs of optima for bimodal gradients.

**Table A3:** Mean *p*‐values of multivariate generalized linear models with standard deviations for combinations of sample size and response type in type I communities

		env1		env2		Noise	
		*μ*	*σ*	*μ*	*σ*	*μ*	*σ*
UU	25	0.002	0.001	0.016	0.005	0.342	0.228
UU	100	0.001	0	0.001	0	0.612	0.267
UU	225	0.001	0	0.001	0	0.697	0.265
UU	400	0.001	0	0.001	0	0.849	0.129
UU	625	0.001	0	0.001	0	0.875	0.138
UU	900	0.001	0	0.001	0	0.801	0.219
UL	25	0.001	0	0.146	0.007	0.781	0.162
UL	100	0.001	0	0.001	0	0.738	0.210
UL	225	0.001	0	0.001	0	0.727	0.283
UL	400	0.001	0	0.001	0	0.729	0.258
UL	625	0.001	0	0.001	0	0.642	0.250
UL	900	0.001	0	0.001	0	0.645	0.272
UB	25	0.022	0.003	0.125	0.010	0.477	0.256
UB	100	0.001	0	0.001	0	0.596	0.264
UB	225	0.001	0	0.001	0	0.737	0.224
UB	400	0.001	0	0.001	0	0.788	0.171
UB	625	0.001	0	0.001	0	0.784	0.249
UB	900	0.001	0	0.001	0	0.811	0.170
LL	25	0.001	0.0004	0.001	0	0.406	0.192
LL	100	0.001	0	0.001	0	0.587	0.277
LL	225	0.001	0	0.001	0	0.514	0.301
LL	400	0.001	0	0.001	0	0.574	0.338
LL	625	0.001	0	0.001	0	0.593	0.319
LL	900	0.001	0	0.001	0	0.460	0.301
LB	25	0.166	0.010	0.001	0	0.776	0.162
LB	100	0.001	0	0.001	0	0.717	0.222
LB	225	0.001	0	0.001	0	0.736	0.285
LB	400	0.001	0	0.001	0	0.721	0.257
LB	625	0.001	0	0.001	0	0.639	0.269
LB	900	0.001	0	0.001	0	0.643	0.275
BB	25	0.001	0	0.010	0.002	0.363	0.242
BB	100	0.001	0	0.001	0	0.432	0.230
BB	225	0.001	0	0.001	0	0.618	0.276
BB	400	0.001	0	0.001	0	0.828	0.158
BB	625	0.001	0	0.001	0	0.814	0.191
BB	900	0.001	0	0.001	0	0.717	0.222

**Table A4:** Mean *p*‐values of constrained quadratic ordination with standard deviations for combinations of sample size and response type in type I communities

		env1		env2		Noise	
		*μ*	*σ*	*μ*	*σ*	*μ*	*σ*
UU	25	0	0	0	0	0.820	0.105
UU	100	0	0	0	0	0.879	0.082
UU	225	0	0	0	0	0.875	0.126
UU	400	0	0	0	0	0.917	0.052
UU	625	0	0	0	0	0.880	0.134
UU	900	0	0	0	0	0.952	0.050
UL	25	0.127	0.158	0.289	0.183	0.646	0.146
UL	100	0.071	0.098	0.240	0.258	0.612	0.267
UL	225	0.004	0.005	0.010	0.012	0.506	0.298
UL	400	0.006	0.013	0.087	0.168	0.569	0.196
UL	625	0	0	0.519	0.269	0.990	0
UL	900	0.008	0.018	0.705	0.401	0.990	0
UB	100	0.024	0.023	0.026	0.026	0.654	0.270
UB	225	0.026	0.058	0.014	0.031	0.702	0.206
UB	400	0.036	0.060	0.002	0.004	0.658	0.274
UB	625	0.010	0.022	0.020	0.044	0.583	0.385
UB	900	0	0	0	0	0.639	0.337
LL	25	0.085	0.048	0.143	0.090	0.400	0.183
LL	100	0.154	0.117	0.095	0.062	0.723	0.176
LL	225	0.091	0.075	0.081	0.060	0.841	0.102
LL	400	0.180	0.137	0.263	0.227	0.761	0.191
LL	625	0.281	0.201	0.208	0.191	0.779	0.126
LL	900	0.295	0.099	0.188	0.146	0.838	0.116
LB	25	0.265	0.127	0.141	0.159	0.619	0.158
LB	100	0.097	0.090	0.038	0.058	0.619	0.278
LB	225	0.067	0.109	0.006	0.013	0.589	0.272
LB	400	0.174	0.237	0.006	0.009	0.586	0.240
LB	625	0.204	0.166	0.044	0.061	0.504	0.260
LB	900	0.164	0.314	0.020	0.028	0.561	0.218
BB	25	0	0	0	0	0.591	0.233
BB	100	0	0	0	0	0.359	0.272
BB	225	0	0	0	0	0.573	0.299
BB	400	0	0	0	0	0.636	0.317
BB	625	0	0	0	0	0.421	0.271
BB	900	0	0	0	0	0.543	0.353

**Table A5:** Mean *p*‐values of canonical correspondence analysis with standard deviations for combinations of sample size and response type in type I communities

		env1		env2		Noise	
		*μ*	*σ*	*μ*	*σ*	*μ*	*σ*
UU	25	0.001	0	0.001	0	0.001	0
UU	100	0.001	0	0.001	0	0.467	0.355
UU	225	0.001	0	0.001	0	0.093	0.113
UU	400	0.001	0	0.001	0	0.349	0.336
UU	625	0.001	0	0.001	0	0.153	0.275
UU	900	0.001	0	0.001	0	0.247	0.362
UL	25	0.001	0	0.987	0.014	0.403	0.288
UL	100	0.001	0	1	0	0.329	0.294
UL	225	0.001	0	1	0	0.346	0.274
UL	400	0.001	0	1	0	0.393	0.317
UL	625	0.001	0	1	0	0.436	0.219
UL	900	0.001	0	1	0	0.439	0.321
UB	25	0.001	0	0.001	0	0.035	0.074
UB	100	0.001	0	0.001	0	0.344	0.269
UB	225	0.001	0	0.001	0	0.244	0.283
UB	400	0.001	0	0.001	0	0.172	0.195
UB	625	0.001	0	0.001	0	0.111	0.192
UB	900	0.001	0	0.001	0	0.066	0.170
LL	25	0.992	0.012	0.997	0.005	0.976	0.031
LL	100	0.985	0.009	0.989	0.005	0.994	0.011
LL	225	0.712	0.046	0.691	0.031	0.962	0.069
LB	25	0.987	0.015	0.001	0	0.398	0.289
LB	100	1	0	0.001	0	0.358	0.307
LB	225	1	0	0.001	0	0.378	0.278
LB	400	1	0	0.001	0	0.412	0.331
LB	625	1	0	0.001	0	0.438	0.209
LB	900	1	0	0.001	0	0.446	0.321
BB	25	0.001	0	0.001	0	0.564	0.286
BB	100	0.001	0	0.001	0	0.469	0.341
BB	225	0.001	0	0.001	0	0.580	0.301
BB	400	0.001	0	0.001	0	0.566	0.314
BB	625	0.001	0	0.001	0	0.497	0.343
BB	900	0.001	0	0.001	0	0.491	0.330

**Table A6:** Mean *p*‐values of distance‐based redundancy analysis with standard deviations for combinations of sample size and response type in type I communities

		env1		env2		Noise	
		*μ*	*σ*	*μ*	*σ*	*μ*	*σ*
UU	25	0.002	0.001	0.001	0	0.511	0.368
UU	100	0.001	0	0.001	0	0.297	0.177
UU	225	0.001	0	0.001	0	0.350	0.227
UU	400	0.001	0	0.001	0	0.503	0.248
UU	625	0.001	0	0.001	0	0.578	0.253
UU	900	0.001	0	0.001	0	0.412	0.305
UL	25	0.001	0	0.055	0.019	0.371	0.342
UL	100	0.001	0	0.001	0	0.444	0.343
UL	225	0.001	0	0.001	0	0.383	0.257
UL	400	0.001	0	0.001	0	0.474	0.271
UL	625	0.001	0	0.001	0	0.396	0.284
UL	900	0.001	0	0.001	0	0.446	0.246
UB	25	0.002	0.001	0.001	0	0.295	0.188
UB	100	0.001	0	0.001	0	0.473	0.290
UB	225	0.001	0	0.001	0	0.394	0.272
UB	400	0.001	0	0.001	0	0.476	0.280
UB	625	0.001	0	0.001	0	0.579	0.167
UB	900	0.001	0	0.001	0	0.403	0.250
LL	25	0.010	0.005	0.009	0.007	0.520	0.219
LL	100	0.001	0	0.001	0	0.467	0.261
LL	225	0.001	0	0.001	0	0.477	0.261
LL	400	0.001	0	0.001	0	0.665	0.293
LL	625	0.001	0	0.001	0	0.589	0.290
LL	900	0.001	0	0.001	0	0.347	0.298
LB	25	0.037	0.016	0.001	0	0.373	0.344
LB	100	0.001	0	0.001	0	0.446	0.342
LB	225	0.001	0	0.001	0	0.396	0.260
LB	400	0.001	0	0.001	0	0.468	0.261
LB	625	0.001	0	0.001	0	0.393	0.284
LB	900	0.001	0	0.001	0	0.429	0.234
BB	25	0.001	0	0.001	0	0.579	0.368
BB	100	0.001	0	0.001	0	0.446	0.280
BB	225	0.001	0	0.001	0	0.472	0.319
BB	400	0.001	0	0.001	0	0.439	0.243
BB	625	0.001	0	0.001	0	0.516	0.269
BB	900	0.001	0	0.001	0	0.397	0.283

**Table A7:** Mean *p*‐values of all four methods with standard deviations for type II communities

	env1		env2		Noise	
	*μ*	*σ*	*μ*	*σ*	*μ*	*σ*
CCA	0.001	0.001	0.001	0.001	0.198	0.198
CQO	0.004	0.004	0.002	0.002	0.648	0.648
dbRDA	0.001	0.001	0.001	0.001	0.311	0.311
MvGLM	0.001	0.001	0.001	0.001	0.589	0.589

**Table A8:** Mean *p*‐values of all four methods with standard deviations for type III communities

	env1		env2		Noise	
	*μ*	*σ*	*μ*	*σ*	*μ*	*σ*
CCA	0.001	0.001	0.001	0.001	0.678	0.678
CCA_Hellinger	0.001	0.001	0.001	0.001	0.431	0.431
CCA_log	0.001	0.001	0.001	0.001	0.583	0.583
CCA_sqrt	0.001	0.001	0.001	0.001	0.642	0.642
CQO	0.008	0.008	0.055	0.055	0.568	0.568
dbRDA	0.001	0.001	0.001	0.001	0.284	0.284
dbRDA_Hellinger	0.001	0.001	0.001	0.001	0.379	0.379
dbRDA_log	0.001	0.001	0.001	0.001	0.317	0.317
dbRDA_sqrt	0.001	0.001	0.001	0.001	0.327	0.327
MvGLM	0.001	0.001	0.001	0.001	0.711	0.711
